# Coordinated prophylactic surgical management for women with hereditary breast-ovarian cancer syndrome

**DOI:** 10.1186/1471-2407-8-101

**Published:** 2008-04-14

**Authors:** Larissa I Batista, Karen H Lu, Elisabeth K Beahm, Banu K Arun, Diane C Bodurka, Funda Meric-Bernstam

**Affiliations:** 1 Department of Surgical Oncology, The University of Texas M. D. Anderson Cancer Center, Houston, TX, USA; 2 Department of Gynecologic Oncology, The University of Texas M. D. Anderson Cancer Center, Houston, TX, USA; 3 Department of Plastic Surgery, The University of Texas M. D. Anderson Cancer Center, Houston, TX, USA; 4 Department of Breast Medical Oncology, The University of Texas M. D. Anderson Cancer Center, Houston, TX, USA

## Abstract

**Background:**

Women with *BRCA1 *or *BRCA2 *mutations have a substantially increased risk of breast and ovarian cancer compared with the general population. Therefore, prophylactic mastectomy (PM) and bilateral salpingo-oophorectomy (BSO) have been proposed as risk-reduction strategies for *BRCA1/2 *mutation carriers. We aimed to assess the feasibility of coordinated PM and BSO in hereditary breast-ovarian cancer syndrome.

**Methods:**

High risk women for breast and ovarian cancer who underwent coordinated PM and BSO were included in this study. Clinical characteristics and surgical and oncologic outcomes were retrospectively reviewed.

**Results:**

Twelve patients underwent coordinated PM and BSO. Ten had history of previous breast cancer. Autologous breast reconstruction was performed in ten patients. The mean age at surgery was 43 (range 34–65). Mean operating time was 9.3 hours (range 3–16) with a mean postoperative hospitalization of 5.4 days (range 4–8). Intraoperatively, there were no major surgical complications. Postoperatively, one patient developed an abdominal wound dehiscence, another reoperation for flap congestion; one had umbilical superficial epidermolysis, and one patient developed aspiration pneumonia. At a mean follow-up of 84 months, 10 of patients were cancer-free. Although no patients developed a new primary cancer, two developed a distant recurrence.

**Conclusion:**

Coordinated PM and BSO is a feasible procedure with acceptable morbidity in selected high-risk patients that desire to undergo surgery at one operative setting.

## Background

Women with *BRCA1 *or *BRCA2 *mutation have a substantially increased risk of breast and ovarian cancer compared with the general population [1]. Breast cancer due to a hereditary cause is about 5 to 10% of all malignant breast disease and 25 to 40% of breast cancers that occur in women younger than 35 years old. Cumulative lifetime risk (to 70 years of age) for invasive breast cancer in women with *BRCA1 *and *BRCA2 *mutation is 40 to 85% and for invasive epithelial ovarian cancer is 15 to 65% [1-4]. Women who are *BRCA1/2 *mutation carriers also have a 26–40% risk of contralateral breast cancer in 10 years [5,6].

Currently, management strategies for high-risk women include intense clinical follow up (every 3–6 months clinical breast exams and annual breast magnetic resonance imaging starting at 25 years of age, as well as gynecologic cancer screening with twice-yearly transvaginal ultrasound and serum CA-125) [7] and chemoprevention with tamoxifen [8,9]. A more aggressive risk reduction approach is PM and BSO. Prophylactic surgery has been shown to be very effective in significantly reducing breast and ovarian cancer occurrence [10-12]. This report describes the feasibility of coordinated PM and BSO in women at high risk for breast-ovarian cancer.

## Methods

### Patient selection

Patients who underwent PM and BSO in the same operation were identified through search of databases maintained by the Department of Medical Informatics and the Department of Gynecologic Oncology. This study was approved by the University of Texas M. D. Anderson Cancer Center Institutional Review Board.

### Surgical technique

The indications for PM and BSO, and other management options were discussed with each patient. Risks, complications and alternatives were discussed and informed consent was obtained. All patients underwent PM and BSO. The patients who were evaluated and were felt to be good candidates for autologous bilateral breast reconstruction from the lower abdominal wall underwent bilateral flap reconstruction of the breast using the transverse rectus abdominus myocutaneous (TRAM) flaps or deep inferior epigastric perforator (DIEP) flaps, and the prophylactic surgeries simultaneously. Patients who did not undergo breast reconstruction had their gynecologic procedure and mastectomy at the same time.

Patients were administered general anesthesia. The breasts and the abdomen were then prepared and draped in the usual manner for exposure of the anterior chest and abdomen. Markings were made along the breast by the reconstructive and breast surgery services. The breast surgical oncology team performed the mastectomy while the abdominal flaps were harvested and transferred by the reconstructive team simultaneously. After that the abdominal flap was inset into the recipient site and revascularized if a free flap was performed. The flaps were temporarily inset and secured to the chest wall while the gynecologic oncology service performed the BSO. The gynecologic team performed the BSO and in some cases, also a total abdominal hysterectomy (TAH). The peritoneum was closed by the gynecologic surgeons and at this point the additional portion of the abdominal closure was carefully performed by the reconstructive team.

### Data Collection and Statistics

Medical records were retrospectively reviewed, and clinical, demographic and genetic characteristics were abstracted. Surgical procedures, operating times, complications and subsequent clinical follow up were also recorded. For patients who had a personal history of previous breast cancer, we also collected data related to disease characteristics and follow up. The results are described as average, standard deviation and percentage.

## Results

From June 1996 to July 2003, twelve high-risk women had coordinated PM and BSO. The mean age at prophylactic surgery was 43 years (range 34–65). The clinical characteristics of the patients are described in Table 1. Eleven of the patients had undergone BRCA testing and were known to have a deleterious mutation. Ten patients had a personal history of breast cancer prior to the prophylactic surgery. Nine patients received chemotherapy, and six received radiation therapy.

**Table 1:** Clinical Characteristics (n = 12)

Characteristics	Number (%)
Mean age at prophylactic surgery (range)	43 (34–65)
BMI (SD)	22.7 ± 3.1*
Race	
White, Non-Ashkenazie Jewish	10 (83.3)
Ashkenazie Jewish	1 (8.3)
Hispanic	1 (8.3)
Personal previous history of breast cancer	10 (83.3)
Primary	9 (75)
Primary with local recurrence	1 (8.3)
Personal history of ovarian cancer	0 (0)
Family history of breast cancer	11 (91.6)
First degree relatives with breast cancer	9 (75)
Family history of ovarian cancer	2 (16.6)
First degree relatives with ovarian cancer	1 (8.3)
Smoking	2 (16.66)
Parity	
1 live birth	2 (16.66)
2 live birth	7 (58.33)
3 live birth	3 (25)
Menopausal Status	
Premenopausal	7 (58.33)
Postmenopausal	5 (41.66)
Due to chemotherapy	1 (8.33)
Due to use of tamoxifen	1 (8.33)
Due to previous hysterectomy	2 (16.66)
Natural menopause	1 (8.33)

*Values are presented as average ± standard deviation

During the prophylactic surgeries, breast and gynecologic surgeries were performed at the same operation. Six patients underwent contralateral mastectomy, four therapeutic/completion mastectomies in addition to the prophylactic contralateral mastectomies and two underwent bilateral prophylactic mastectomy. All patients underwent open BSO and seven also underwent a total abdominal hysterectomy (TAH). Ten patients underwent bilateral reconstruction, nine with TRAM flap and one with DIEP flap. The indication and technical details of the coordinated surgical prophylactic and complementary procedures are detailed in Table 2.

**Table 2:** Coordinated Surgical Procedures and Complications

Patient	Previous Breast Cancer	Prophylactic Breast Surgery	Prophylactic Gynecologic Surgery	Reconstruction	Postoperative Complications
1	yes	Contralateral mastectomy	BSO (previous TAH)	Free TRAM	None
2	no	Bilateral mastectomy	TAH-BSO	Pedicle TRAM	None
3	yes	Contralateral mastectomy	BSO (previous TAH)	no	None
4	yes	Contralateral mastectomy	TAH-BSO	no	None
5	yes	Contralateral mastectomy	BSO	Pedicle TRAM	Reoperation for flap congestion
6	yes	Contralateral mastectomy	TAH-BSO	Free TRAM	Superficial epidermolysis of the umbilicus
7	yes	Contralateral + Completion mastectomy	TAH-BSO	Free TRAM	None
8	yes	Contralateral mastectomy	BSO	Free DIEP	None
9	no	Bilateral mastectomy	TAH-BSO	Free TRAM	None
10	yes	Contralateral mastectomy	TAH-BSO	Free TRAM	Postoperative aspiration pneumonia
11	yes	Contralateral + Completion mastectomy	BSO (previous TAH)	Free TRAM	None
12	yes	Contralateral + Completion mastectomy	TAH-BSO	Free TRAM	Abdominal wound dehiscence

BSO = Bilateral salpingo-oophorectomy.

TAH = Total abdominal hysterectomy.

TRAM = Transverse rectus abdominus myocutaneous.

DIEP = Deep inferior epigastric perforator.

The mean operating time was 9.3 hours (range 3 – 16) with a hospital stay of 5.4 days (range 4–8). There were no cases of death, bleeding requiring blood transfusion or other major complication during surgery.

The postoperative complications seen are listed in the Table 2. In one patient a large ventral hernia was found during the TRAM flap dissection, which was repaired with polyproprolene mesh at that time. Postoperatively, this patient had an abdominal wound dehiscence. The abdominal wall (fascia and mesh) remained intact. The wound was managed conservatively and completely closed about 1 month later with no further complications. A patient who underwent reconstruction with pedicle TRAM flaps, right delayed, left immediate had a reoperation for left flap congestion. A super-charge of left pedicle TRAM flap, artery and vein, was successfully performed. Placement with bilateral saline-filled mammary implants was performed one year later due to a contour deformity of the superior pole of the breast mound. Another patient who had prophylactic contralateral mastectomy and TAH-BSO followed by reconstruction of both breasts with bilateral free TRAM flap, had some superficial epidermolysis of her umbilicus postoperatively. This was managed conservatively, leaving a hypertrophic scar with no further complications. The development of aspiration pneumonia was seen postoperatively in one patient who underwent contralateral prophylactic mastectomy, TAH-BSO, and free TRAM reconstruction. This was treated aggressively with pulmonary physiotherapy and intravenous antibiotics. Otherwise, the postoperative course was uneventful. Even with those complications, all patients recovered well postoperatively. There were no complications directly attributable to the combination of the gynecologic and breast procedures.

At a mean follow-up was 84 months, none of the patients developed a new breast cancer. However, two patients died due to breast cancer metastasis; both patients had a breast cancer diagnosis at the time of their risk-reducing surgery. The first patient was diagnosed with breast cancer at 29 years of age with a clinical stage IIIA breast cancer. She underwent prophylactic contralateral mastectomy and BSO one year after the diagnosis. She was free of disease for 2 years when was found to have bone, lung and brain metastasis. She received palliative chemotherapy but died from breast cancer nine months later. The second one was 31 when was diagnosed with a Stage IIA breast cancer. She underwent prophylactic contralateral mastectomy and BSO about 2 years after the diagnosis. Ten months after prophylactic surgery, she was found to have metastatic disease to the brain. She received palliative chemotherapy and deceased four and a half years later. Both patients had undergone a negative staging work-up with a chest x-ray, bone scan and abdominal CT at their initial breast cancer diagnosis (11 and 15 months prior to prophylactic surgery). The remaining 10 patients did not have recurrence and are still alive.

## Discussion

Several studies have shown that the risk of breast and ovarian cancer can be decreased by prophylactic surgery in carriers of *BRCA *mutations. Prophylactic BSO not only reduces the risk of ovarian cancer by 80–95% but also reduces the risk of contralateral breast cancer by 50% [10,13]. Rebbeck et al demonstrated that bilateral PM reduced the risk of breast cancer by 90% in women with intact ovaries and by 95% in women who underwent both PM and oophorectomy [12]. Our study aimed to evaluate the feasibility of coordinated prophylactic mastectomy and BSO in high risk breast-ovarian cancer women.

Women with breast cancer who carry deleterious *BRCA *mutations are at increased risk of developing a second primary breast cancer as well as a primary ovarian cancer [14]. However, the prevention of a second primary breast cancer by prophylactic mastectomy may be overshadowed by the prognosis of the first tumor. In a decision analysis, Schrag et al demonstrated that life expectancy gains from risk-reducing surgery is greatest for patients that are young, for high-penetrance mutations, and in node-negative disease [15]. The breast cancer-related deaths of two of the patients in our small series underscore the challenges of patient selection for risk-reducing procedures.

It is critical to discuss immediate breast reconstruction with all patients undergoing PM. Most patients undergoing bilateral PM for risk-reduction are candidates for reconstruction. Immediate reconstruction is also usually feasible in most patients undergoing a mastectomy for breast cancer, but may be deferred in patients in whom postmastectomy radiation therapy is planned [16,17] Reconstructive options include implant-based reconstruction, as well as autologous reconstruction approaches such as pedicled TRAM flap, or free TRAM flap with various degrees of muscle sparing, the deep inferior epigastric artery perforator (DIEP) and the superficial inferior epigastric artery (SIEA) flap. Implant-based reconstruction is preferred by some due to ease of performance, while autologous tissue reconstruction is preferred by others due to its natural shape, soft consistency and long-lasting aesthetic results. The choice of reconstruction is an important determinant of operative time, and potential morbidity of the surgery. Most patients who elect contralateral prophylactic mastectomy (CPM) at our institution undergo autologous tissue reconstruction [18]. The choice of reconstruction is an important determinant of operative time, and potential morbidity of the surgery. At our institution, with the use of free TRAM flap reconstruction, flap complications have been observed in 24% of patients, and donor site complications in 15% of patients [19]. In this series, 10 of 12 patients underwent reconstruction with abdominal TRAM flaps, and even though the reconstruction increased the operative time, there were no major intraoperative or postoperative complications. The complications seen in this study are in line with our previous institutional experience with mastectomy and breast reconstruction [19]. Thus in this limited series, adding the gynecologic surgery to the breast procedure resulted in acceptable morbidity.

Although we did not have any substantial complications associated with the gynecologic surgery in our series, there is a potential risk in adding the gynecologic procedure to prophylactic surgery, especially in the setting of autologous reconstructive surgery. There is a small risk of intra-abdominal bleeding, which could lead to a low flow state for the autologous flap, and in the case of a microvascular free flap, even to flap loss. Hysterectomy exposes the abdomen to vaginal bacterial flora and may also increase the risk of wound infections and other wound complications, a risk factor for infection and loss of tissue-expander/implant-based reconstruction. The magnitude of a bilateral autologous breast reconstruction is significant for both patient and surgeon, and the impact of flap loss, while rare (less than 2% in our institution), is profound. For gynecologic procedures, the patient is placed in Trendelenberg immediately after revascularization of the flap, the most vulnerable time period for vascular compromise. In addition, the flap can not be easily visualized at this time. There are accordant risks for flap avulsion or compromise of the vascular pedicle, especially in the newer perforator based flaps, such as the DIEP flap, which were developed to spare the abdominal donor site morbidity associated with increased muscle harvest of the rectus abdominis muscle, but leave the vascular pedicle much less protected. Additionally, there is need for careful closure of the abdominal donor site to prevent risk of herniation.

Coordinated single operation has three main advantages. The first is that it allows for a single operation and recovery, potentially enhancing patient convenience. The second is that oophorectomy may allow for the initiation of aromatase inhibitors for endocrine treatment in premenopausal patients with estrogen-receptor positive disease. The third is that it allows for early ovarian risk reduction, minimizing the theoretical risk of ovarian cancer development in between staged procedures. These advantages of a single operation should be weighed against potential morbidity of adding the gynecological surgery to the prophylactic mastectomy with reconstruction. As an alternative,

As breast reconstruction is a staged procedure involving a series of 2–3 procedures several months apart, the gynecologic procedure may be performed during one of the secondary reconstructive procedures.

In our series of patients, seven of nine patients who had a uterus at that time also underwent TAH during the procedure. No complications due to hysterectomy were seen. However, our group and others are moving away from performing hysterectomy routinely in these patients. In patients with uterine or cervical abnormalities, hysterectomy may be considered. In addition, women who have taken tamoxifen may consider hysterectomy due to the increased risk of endometrial cancer. However, patients need to be counseled that the addition of hysterectomy to bilateral oophorectomy may increase surgical time and morbidity [20].

Our study is limited as it is a small retrospective case series from a single institution. Further, our center has significant expertise in breast and gynecologic oncology as well as reconstructive surgery; this coordinated approach may be met with additional challenges, including higher rates of morbidity, when performed by lower volume surgical teams. Finally, due to our short clinical follow-up and small sample size, we are unable to report long-term risk reduction rates.

## Conclusion

In conclusion, our study showed that coordinated PM and BSO is a feasible procedure with acceptable morbidity for patients at high risk for breast and ovarian cancer who elect to undergo synchronous risk-reduction operations. However, the optimal approach and timing for risk-reducing surgery in women at high-risk for breast-ovarian cancer needs to be determined in a multidisciplinary fashion, taking into account several factors including the time-line of greatest breast and gynecologic cancer risk and also the potential complications of the operations.

## Competing interests

The author(s) declare that they have no competing interests.

## Authors' contributions

LB was responsible for data collection data analysis and writing of the manuscript. KL was responsible for conception, data collection, data analysis and editing of the manuscript. EB was responsible for assistance in data collection, data analysis and editing of the final manuscript. BA was responsible for editing the final manuscript. DB was responsible for conception and editing the final manuscript. FMB was responsible for conception, assistance in study design, final data analysis, writing and finalizing the manuscript. All authors have read and approved the final manuscript.

## Pre-publication history

The pre-publication history for this paper can be accessed here:


